# Effect of erosive solutions and thermal cycling on the surface properties of universal injectable and regular consistency resin composites

**DOI:** 10.1186/s12903-025-06950-y

**Published:** 2025-10-02

**Authors:** Ahmed Abbas Rhaif, Hoda Saleh Ismail, Tawakol Ahmed Ahmed Enab, Nadia Mohamed Zaghloul

**Affiliations:** 1 Conservative Dentistry Department, Faculty of Dentistry, Mansoura University, 35516, Algomhoria Street, Mansoura, Aldakhlia Egypt; 2 Department of Production and Mechanical Design, Faculty of Engineering, Mansoura University, Mansoura, Egypt

**Keywords:** Universal injectable composites, Surface roughness, Microhardness, Wear resistance, Dental erosion

## Abstract

**Background:**

Injectable resin composites, such as G-aenial Universal and Bulk Injectable, offer high flowability and easy application for minimally invasive repair of eroded or chipped teeth. However, limited in vitro data exist regarding their resistance to erosive and thermal aging under neutral and acidic oral conditions.

**Materials and methods:**

Disc-shaped specimens (10 mm × 2 mm) were prepared from two injectable composites—G-aenial Universal Injectable and G-aenial Bulk Injectable—and two conventional composites—3 M Filtek Z350 XT Universal Restorative and 3 M Filtek One Bulkfill. Seventy-two discs per material were subdivided into three aging protocols (*n* = 24 each): (1) deionized water immersion (pH 7), (2) HCl immersion (pH 1.2) followed by thermal cycling, or (3) citric acid immersion (pH 3) followed by thermal cycling. Each subgroup was tested for surface roughness, Vickers microhardness, and two-body wear resistance (*n* = 8). Surface roughness was measured via contact profilometry; selected specimens underwent atomic force microscope (AFM) and scanning electron microscope (SEM) imaging. Microhardness was assessed using a digital Vickers tester, and wear resistance was simulated with a programmable wear device measuring weight and volume loss. Two-way ANOVA, post-hoc, and Pearson’s correlation analyses were performed (*p* < 0.05).

**Results:**

Erosive and thermal aging led to significant increases in surface roughness and reductions in microhardness across most materials, with no significant change in two-body wear resistance. Under the HCl + thermal cycling condition, G-aenial Bulk Injectable demonstrated the highest roughness and lowest hardness, whereas Filtek Z350 XT Universal showed the lowest roughness and highest hardness. Following citric acid exposure, no significant differences in roughness or hardness were identified, although G-aenial Bulk Injectable incurred the greatest weight loss.

**Conclusions:**

According to this in vitro study, G-aenial Universal Injectable is best suited for neutral pH oral conditions, while Filtek Z350 XT Universal provides superior performance under acidic challenge. Incremental composites generally preserve surface quality better than bulk-fill materials. The more severe degradation resulting from gastric-level acid compared to citric acid illustrates the value of selecting restorative composites based on anticipated oral environmental exposures.

## Introduction

Resin-based composites are frequently selected in restorative dentistry because of their superior aesthetics and mechanical properties. Advanced filler technology, particularly nanofillers and nanoclusters, has improved strength, wear resistance, gloss retention, and polishability while minimizing polymerization shrinkage [1, 2]. Flowable composites include a reduced amount of filler and an increased proportion of resin modifiers, enhancing their adaptability to cavities while compromising their mechanical durability [3, 4]. To address this issue, injectable composites, characterized by high filler density and low viscosity, currently possess mechanical and physical qualities similar to those of conventional paste composites [5]. Bulkfill composites were developed to enhance efficiency in posterior restorations by allowing 4–5 mm single-increment application. Improved translucency, optimized photoinitiators, and customized filler systems enable these materials to attain sufficient depth of cure, surface hardness, and wear resistance comparable to conventional incremental methods [6, 7].

Despite advancements in these materials, acidic challenges like vomiting, GERD, and dietary acids can compromise resin composites and enamel by increasing acidity, degrading bonding, corroding coatings, and inducing wear, resulting in diminished hardness, roughened surfaces, and reduced strength. Temperature changes speed up the aging of composite materials in wet conditions by causing internal stress because the matrix and filler expand at different rates, which leads to rougher surfaces and lower hardness and fatigue resistance [8–19].

Surface roughness influences gloss, stain resistance, and plaque accumulation. Bacteria can inhabit surfaces above 0.2 μm, whereas patients may observe levels beyond 0.3 μm, hence increasing the risk of periodontitis and subsequent caries [13, 20, 21]. Microhardness, defined as a material’s resistance to indentation, indicates conversion efficiency and corresponds with strength, wear resistance, and chipping resistance, as accurately assessed by Vickers testing; inadequate hardness increases the danger of fracture [22–26]. Wear resistance is essential for durability under occlusal forces, especially in parafunctional behaviors; it is influenced by elements such as abrasive food, opposing materials, and composite filler properties. Advancements in filler morphology, particularly the incorporation of nanofillers, enhance fatigue, abrasive, and corrosive wear resistance by fortifying the resin matrix and restricting fracture propagation [27–31].

Since “universal injectable composites” is a new category and there is little research on how different erosive solutions affect these materials, more studies are needed. This study intends to address the knowledge gap regarding patients with erosive tooth wear who may not dramatically modify their lifestyle or behavior after receiving restorations. This study aimed to evaluate and compare how erosive solutions and temperature changes affect the surface roughness, hardness, and wear resistance of universal injectable composites and regular composites, including both layered and bulkfill types. The null hypotheses examined were as follows: 1) No significant variations were seen in roughness, microhardness, and wear resistance among the evaluated restorative materials, regardless of the ageing process. The ageing conditions would not influence the roughness, microhardness, or wear resistance of the evaluated restorative materials.

## Materials and methods

This study employed four distinct resin composite materials, each distinguished by their respective brands. The materials used were as follows: G-aenial Universal Injectable (G-aenial Universal), G-aenial Bulk Injectable (G-aenial Bulk), 3 M Filtek Z350 XT Universal Restorative (3 M Filtek Universal), and 3 M Filtek One Bulkfill Restorative (3 M Filtek Bulk). The attributes considered for each material included shade, specification, manufacturer, composition, application technique, and lot number, as shown in Table 1.

**Table 1:** Restorative materials used in the study

Restorative materials	Shade	Specification	Manufacturer	Composition	Application technique	Lot number
G-aenial Universal Injectable **(**G-aenial Universal**)**	A2	Incremental nanofilled, universal injectable resin composites	GC Corporation, Tokyo, Japan.	**Matrix**: (31%) in weight Bis-GMA, Bis-EMA, methacrylate monomer **Fillers**: (16 nm silica and 150 nm barium glass) make up 69% in weight(wt.). Ultra-fine particles combined with a silane coupling agent serve as a filler. Pigment, photoinitiator, barium glass, strontium glass, and silicon dioxide.	1-Turn the syringe counter clockwise to remove the cap while holding it upright. 2- Keeping the syringe upright, take off the cap by turning it. 3- The dispensing tip should be positioned as close to the mold as possible. Then gradually press the plunger to force the material out. 4- Light curing for 20 s at a minimum intensity of > 700 mW/cm^2^. The light guide tip was kept in direct contact with the glass slide.	2,305,101
G-aenial Bulk Injectable (G-aenial Bulk)	A2	Bulkfill nanofilled, universal injectable resin composites	GC Corporation	**Matrix**: (Bis-EMA) ** 10 - < 25% wt., dimethacrylate component **2.5 - < 5% wt., bismethacrylate** 2.5 - < 5% wt., and (UDMA)** 0.2 - < 0.5% wt. **Fillers**: UV-light absorber**, ultrafine barium-glass particles formula (150 nm particle size, 69% wt. filler loading).	1. Holding the syringe upright, turn it counter clockwise to remove the cap. 2. Promotion and security: spin the syringe clockwise to secure the dispensing tip. 3. Set the light-protective cap in place until needed. 4. Take off the dispensing tip’s cap. 5. Position the dispensing point as close to the mold as you can. Then gradually press the plunger to force the material out, and cure it. 6. Light curing for 30 s at a minimum intensity of 700–1200 mW/cm^2^, keeping the light guide as close to the surface as feasible.	2,310,061
3 M Filtek Z350 XT Universal Restorative (3 M Filtek Universal)	A2B	Incremental nanofilled, regular consistency resin composites	3 M ESPE Dental Products; St. Paul, MN, USA.	**Matrix**: Bis-GMA, UDMA, TEGDMA, PEGDMA, and Bis-EMA. **Fillers**: a blend of 4–11 nm non-agglomerated zirconia, 20 nm non-agglomerated/non-aggregated silica, and an agglomerated zirconia/silica clusters filler (consisting of 4–11 nm zirconia particles and 20 nm silica). Cluster particles typically range in size from 0.6 to 10 microns. (63.3% by volume and 78.5% wt. of payload).	1. Condense the resin composites to fit the mold surfaces and apply them in small increments. 2. Light cure for 20 s at a minimum intensity of 550–1000 mW/cm^2^. Light guides should be positioned as near to the composites’ surface as feasible. Maintain the light guidance as near the surface as you can.	9,456,930
3 M Filtek One Bulkfill Restorative (3 M Filtek Bulk)	A2	Bulkfill nanofilled, regular consistency resin composites	3 M ESPE Dental Products	**Matrix**: 1,12-dodecanDMA, diurethane-DMA, addition-fragmentation monomers, and AUDMA. **Fillers**: a mixture of 20 nm non-agglomerated/non aggregated silica filler, 4–11 nm non-agglomerated zirconia filler, and an agglomerated zirconia/silica clusters filler (made up of 20 nm silica and 4–11 nm zirconia particles), as well as a 100 nm agglomerate particle ytterbium trifluoride filler. About 76% by wt. of the loading is inorganic (58.5% by volume).	1. Insert the resin composites into the mold, compress it to fit the mold surfaces, and then light cure them in one bulk increment. 2. Light cure for 20 s at a minimum intensity of 550–1000 mW/cm^2^. Light guides should be positioned as close to the composites’ surface as possible. Maintain the light guidance as close to the surface as you can.	9,979,065

A material is considered a trade secret if it has the ** symbol

*Bis-GMA* or bisphenol A-glycidyl methacrylate, *Bis-EMA* or ethoxylated bisphenol A dimethacrylate, *UDMA* or urethane dimethacrylate, *TEGDMA* or triethylene glycol dimethacrylate, *DMA* or dimethacrylate, *PEGDMA* or polyethylene glycol dimethacrylate, *AUDMA* or aromatic urethane dimethacrylate

### Sample size calculation

The preceding study employed GPower software to determine the sample size for roughness and microhardness assessments. The mean and standard deviation data for G-aenial Universal Injectable and Filtek Supreme XTE Flowable Restorative were used for the calculation. The parameters analysed were: a two-tailed test, an effect size of 2.59, a significance threshold of 0.05, 90% power, and an allocation ratio of 1. The initially calculated sample size per subgroup was five, but it was augmented to eight to accommodate variations in the study design and the control material utilised in the cited study. The identical methodology was utilised for microhardness assessments, employing mean and standard deviation data from G-aenial Universal Injectable and Filtek Bulkfill Flowable Restorative. The determined sample size for each subgroup was eight [32]. Furthermore, the earlier study used average and standard deviation information about the wear of G-aenial Universal Injectable and Cerasmart to figure out how many specimens were needed for the wear resistance test. The specifications were a two-tailed test, an effect size of 2.21, a significance threshold of 0.05, 90% power, and an allocation ratio of 1. The initially calculated sample size per subgroup was six; however, due to variations in study design and the use of indirect composites as the control material, it was augmented to eight [33].

### Study design

**Fig. 1:**
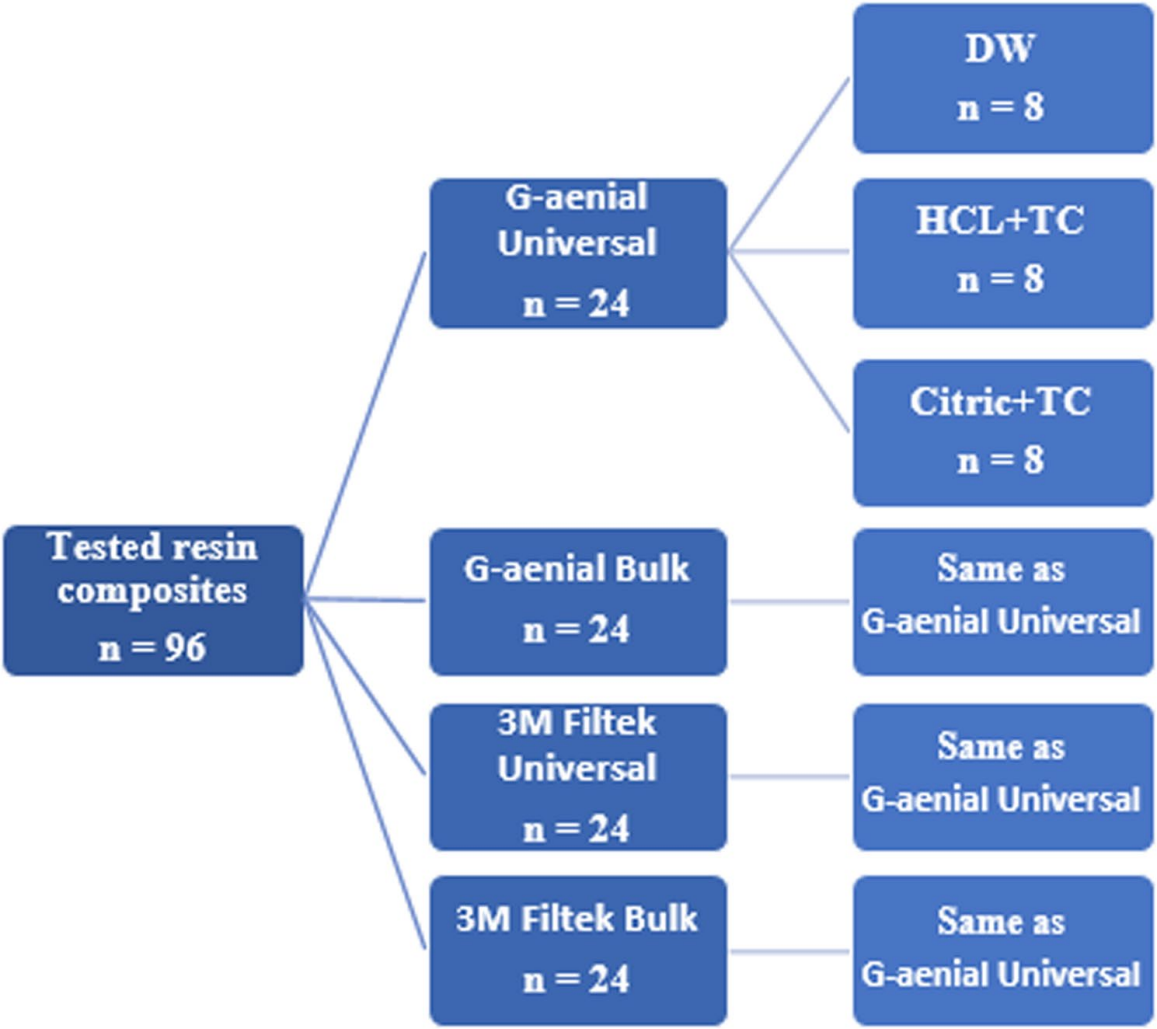
Flowchart showing study design diagram for each of the tests employed

A total of 96 disc-shaped specimens were prepared for each of the three tests performed in this study. These specimens were divided into four material-based groups (*n* = 24 each): Group 1: G-aenial Universal, Group 2: G-aenial Bulk, Group 3: 3 M Filtek Universal, Group 4: 3 M Filtek Bulk. Each group was further subdivided into three aging condition subgroups (*n* = 8 per subgroup): Subgroup a deionized water (DW): Stored in deionized water for 24 h, Subgroup b (HCL + TC): Immersed in HCL solution and then subjected to 10,000 thermal cycles, Subgroup c (Citric + TC): Immersed in citric acid solution followed by 10,000 thermal cycles. These subgroups provided the specimens used in the surface roughness, microhardness, and wear resistance tests. This design allows direct comparison of material performance across controlled aging conditions. A detailed depiction of the study design is presented in Fig. 1.

### Specimen preparation

The experiment involved preparing 288-disc specimens of resin composite using a cylindrical plastic split mold with a central hole measuring 10 mm in diameter and 2 mm in depth. A glass slide and a mylar strip were placed beneath the mold, and for 3 M Filtek Universal and 3 M Filtek Bulk, a gold-plated tool was used to apply the resin. For G-aenial Universal and G-aenial Bulk, a needle tip was placed on the mylar strip to prevent air bubble entrapment. The mold was then covered with a mylar strip and a glass slide, and constant pressure was applied to minimize imperfections and voids on the resin composite surface. The resin composite specimens were cured using a polywave light-curing unit (Curingpen-E, Eighteeth, China) following the manufacturer’s guidelines, plus an extra 20 s of light on the bottom side. The specimens were thoroughly washed with distilled water and cleaned in a BioSonic UC100 (Coltene, Switzerland) ultrasonic bath for ten minutes. Next, each specimen was marked for identification and smoothed using aluminum oxide discs (Sof-Lex, 3 M Espe, USA) in a consistent direction.

### Erosive solution & thermal cycling

Each resin composite group (*n* = 72) was separated into three aging subgroups (*n* = 24), with each subgroup further divided into eight specimens for surface analysis: DW: specimens were incubated in DW (pH 7) at 37 °C for 24 h. Table 2 illustrates that intrinsic erosion (HCL): specimens immersed in 2.16% HCL (pH = 1.2) for 3 h, with hourly rinses and solution renewal to avert contamination, accurately replicate the erosive effects of stomach exposure on composite surfaces over the course of one year [34, 35] Specimens soaked in 5% citric acid (pH = 3) for 2 weeks, with daily changes of the solution and rinses, and then another 2 weeks in the same citric acid solution, have been widely used to imitate how acidic foods wear down enamel and age composites over a year. Citric acid in comparable amounts has been extensively utilized to simulate enamel degradation and composite ageing by mimicking the cumulative effects of acidic meals over a year [36]. Thereafter, specimens from the HCL and Citric ageing subgroups were subjected to 10,000 heat cycles ranging from 5 °C to 55 °C (25 s dwell time, 10 s transfer time) utilizing the ROBOTA automated thermal cycler (BILGE, Turkey), thereby imitating roughly one year of oral temperature variations [37].

**Table 2:** Aging protocol used in the study

Aging Protocol	Simulation Target	Exposure Time
HCL, pH 1.2	Intrinsic (HCL)	3 h
Citric acid, pH 3	Extrinsic (Citric acid)	2 weeks
Thermal Cyclin	Oral temperature fluctuations	—

### Measurement of surface roughness

#### Stylus profilometer evaluation

A profilometer (Mitutoyo, Model No. SJ-210, Mitutoyo America Corporation, USA) was employed to analyze all resin composite discs. A diamond stylus with a tip radius of 5 μm and a tip angle of 90° was utilized. The surface roughness cut-off value was set at 0.25 mm, with the diamond stylus tracing a length of 5.6 mm at a constant speed of 0.5 mm/second. For reliable results, each specimen underwent multiple measurements at the center of the specimen, moving the profilometer parallelly by 0.5 mm for each measurement. Roughness evaluation was conducted in the opposite direction to the finishing/polishing direction. The mean roughness parameters (Ra) and (Rz) values in micrometers (µm) were calculated as the average value. The software (Mitutoyo Ver. 4.00) is utilized for analyzing and manipulating the recorded data.

#### AFM and SEM evaluation

Two representative specimens with roughness values close to the mean were selected from each composite, with one designated for surface analysis using (AFM) and the other for (SEM). An atomic force microscope (Model FlexAFM3, Nanosurf AG, Switzerland) was employed to examine the specimens in the region previously assessed by a profilometer. The tapping mode was used to scan a 50 × 50 μm² area with 256 × 256 data points at a rate of 1 Hz. Images in both height-mode and deflection were simultaneously captured at a resolution of 512 × 512 pixels. To convert pixel measurements to real-world units, the device was calibrated by comparing software-generated scale with a known-size object. Subsequently, a 3D image of the specimen’s surface profile was generated. Data analysis was performed using specialized software (Nanosurf C3000 version 3.5.0.31 software). The finished or polished surfaces of the specimens were prepared for SEM (JSM-6510LV, JEOL Ltd., Japan) by two rounds of gold sputtering (SPI Module Sputter Carbon/Gold Coater, EDEN Instruments, Tokyo, Japan) with a current of 10 mA and a vacuum of 130 mTorr. Images displayed on a computer screen, providing a three-dimensional representation of the material at magnifications of ×250, ×500, ×1,000, and ×2,000.

### Microhardness test

The Vickers hardness number (VHN) of the specimens was measured using a digital Vickers microhardness tester (Model HVS-50, Laizhou Huayin Testing Instrument Co., Ltd., China) [38]. This measurement was carried out with a Vickers diamond indenter at magnification ×20 using an objective lens to quantify the surface microhardness of the specimens. A 100-gram weight was applied to each specimen’s surface for a duration of 15 s [39]. Three indentations were made on the surface of every specimen. The indentations were located in the center of each specimen, 2 mm from the border, and at least 0.5 mm apart from one another [38]. A built-in scaled microscope was utilized to measure the diagonal lengths of the indentations, and these Vickers values were converted into microhardness values. The average of the three measurements obtained from each specimen was calculated to provide the VHN readings. The Vickers number was computed utilizing the formula VHN = 1.854 (P/D²).

where 1.854 was a constant value, P was the load applied in (g), D² was the diagonal average length in (µm²), and VHN was the Vickers hardness in (g/µm²) [40].

### Wear resistance test

Two-body wear testing was conducted using the ROBOTA programmable chewing simulator (Model ACH-09075DC-T, AdTech Technology Co., Germany), a four-station, dual-axis device driven by a servomotor with integrated thermal cycling. Opposing enamel antagonists were prepared from sectioned extracted premolars. Ethical approval was granted by the Ethics Committee (ID: A02012023CD); teeth were obtained from patients undergoing routine orthodontic treatment and periodontal surgery, following their informed consent for use in research, and mounted in a screw-tightened “Jacob’s chuck.” Specimens (10 mm × 2 mm) were subjected to 150,000 cycles (700 g/7 N force) at 5–55 °C with 10-second dwell times to simulate approximately one year of mastication [41]. After testing, specimens were rinsed, ultrasonically cleaned for 10 min, and then assessed for wear. (Table 3.)

**Table 3.** Showing the ROBOTA chewing simulator device's wear test parameters

Vertical movement: 1 mm	Horizontal movement: 5 mm
Rising speed: 90 mm/s	Forward speed: 90 mm/s
Descending speed: 40 mm/s	Backward speed: 40 mm/s
Cycle frequency 1.6 Hz	Weight per specimen: from 700 gr

Torque; 2.4 N.m

### Wear measurements

#### Weight loss measurement

Weight measurements were taken before the wear stages to determine the initial specimen weight. Post-loading weight loss was used to calculate the substance loss of the specimens. Electronic analytical scale (AS Solid Lab Analytical Balance 0.1 Mg; Sartorius; Germany) with a precision of 0.0001 g, recording differences before and after each wear cycle. The electronic balance used for measuring the resin composite disc and antagonist specimens had a small weighing scale and automatic calibration features to ensure accurate measurements. Before weighing, each mounted specimen was cleaned and dried with tissue paper. The balance’s glass doors were shut to prevent air disturbances, and it was placed on a stable table to avoid vibrations and ensure accuracy [42]. The following equation [43] was used to get the final mass (M2) for each Specimen and to report the mass loss for each material after abrasion:

W% = ([M2 − M1]/M1) × 100% M1 = initial mass before abrasion.

#### Volume loss measurement

The quantitative analysis of two-body wear on the specimens was performed before and after loading using a 3D surface analyzer system. Following the wear testing, the specimens were immediately cleaned with distilled water to remove any debris. Random wear scar profiles were selected from the center of the abrasion area, perpendicular to the direction of sliding. From these profiles, the wear scar’s length and depth were then determined [9]. The dimensions of the wear scar were measured using a USB digital microscope with an integrated camera (Scope Capture Digital Microscope, Guangdong, China) connected to an IBM-compatible personal computer at a fixed magnification of ×120. The entire surface of the wear scar was scanned. Three points on the unworn surface were selected to establish a reference plane for the dimension measurements. Specialized software, the WSxM software (Ver 5 build 4.1, Nanotec, Electronica SL, Spain), was employed to calculate the volume loss of the specimens [9]. This software served as a 3D image processing program to overlay the 3D models before and after the wear simulation and perform a subtraction operation to determine the volumetric loss [44].

### Statistical analysis

Data were analyzed using SPSS^®^ software version 25 (SPSS Inc., Chicago, IL, USA). The Shapiro–Wilk test confirmed normal distribution for all variables. As the data were parametric, descriptive statistics were reported as means and standard deviations. A two-way ANOVA was used to evaluate the effects of the composite type and aging condition on surface roughness (Ra, Rz), microhardness, volume loss, and weight loss. When significant effects were detected, Bonferroni-corrected post hoc comparisons were applied. Pearson correlation coefficients were calculated to explore relationships between the tested parameters. A p-value < 0.05 was considered statistically significant.

## Results

### Roughness results by stylus profilometer

Table 4 shows the average surface roughness values (Ra and Rz), along with their standard deviations (SDs) and 95% confidence intervals (CIs), for each composite under various aging conditions. Two-way ANOVA showed that both the type of composite and the aging condition had a significant impact, and there was also a notable interaction between the two (Ra: *p* < 0.001; Rz: *p* < 0.001).

**Table 4:** Presents a comparison of roughness (Ra, Rz) (µm) across several composite types and aging conditions, including averages, standard deviations (SD), and p-values

Contact Ra, Rz (µm)	DW	HCL + TC	Citric + TC	DW–HCL + TC	DW-Citric + TC	HCL + TC-Citric + TC
	Mean, (SD)	Mean, (SD)	Mean, (SD)			
	Ra, Rz	Ra, Rz	Ra, Rz	Ra, Rz	Ra, Rz	Ra, Rz
G-aenial Universal	0.070^A^, 0.431^A^, (0.027, 0.117)	0.152^A^, 0.903 ^A^, (0.097, 0.568)	0.082 ^A^, 0.443^A^, (0.030, 0.153)	0.009*,0.009*	0.693,0.947	0.026*,0.011*
G-aenial Bulk	0.108 ^A^, 0.499 ^A^, (0.047, 0.175)	0.443^B^, 1.653^B^, (0.279 *, 1.448)	0.088 ^A^, 0.476 ^A^, (0.075, 0.338)	< 0.001*, < 0.001*	0.531,0.902	< 0.001*, < 0.001*
3 M Filtek Universal	0.082 ^A^, 0.524 ^A^, (0.025, 0.131)	0.088^C^, 0.547^C^, (0.036 *, 0.237)	0.063 ^A^, 0.415 ^A^ (0.023, 0.130)	0.835,0.898	0.503,0.536	0.381,0.461
3 M Filtek Bulk	0.061 ^A^, 0.399 ^A^, (0.015, 0.077)	0.163 ^A^, 1.040 ^A^, (0.050, 0.498)	0.062 ^A^, 0.434 ^A^, (0.011, 0.077)	0.001*, < 0.001*	0.948,0.864	0.001*,0.001*

p* is significant at the 5% level. Distinct letters inside the same column indicated a statistically significant difference between each pair of groups (Bonferroni test, p<0.05). Identical letters inside the same column indicated no statistically significant difference between the two groups (Bonferroni test, p> 0.05) in all following tables

*X* mean, *SD* Standard deviation

#### Composite type effect

**Fig. 2:**
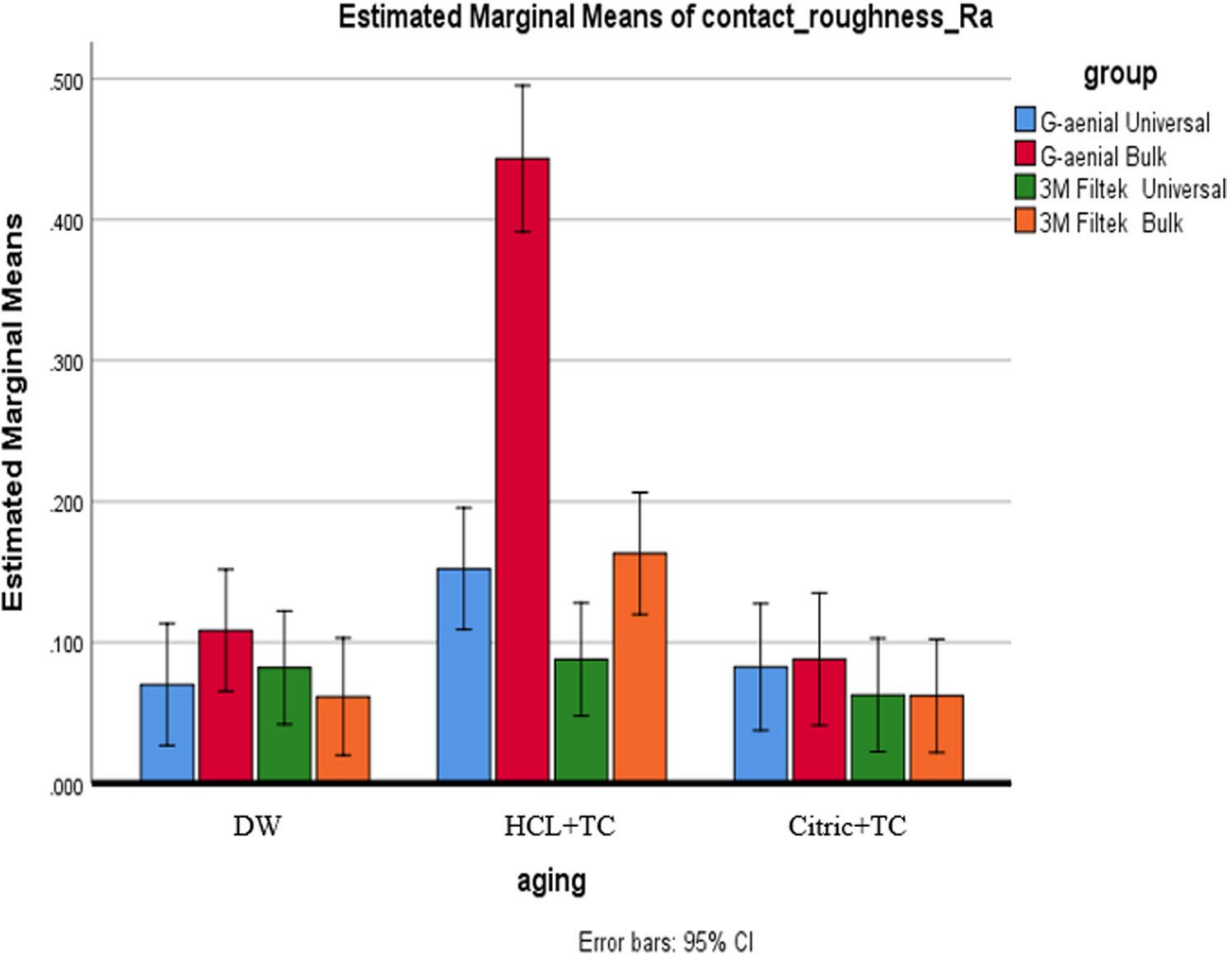
Presents a comparison of Ra between various types of composites under each aging condition

**Fig. 3:**
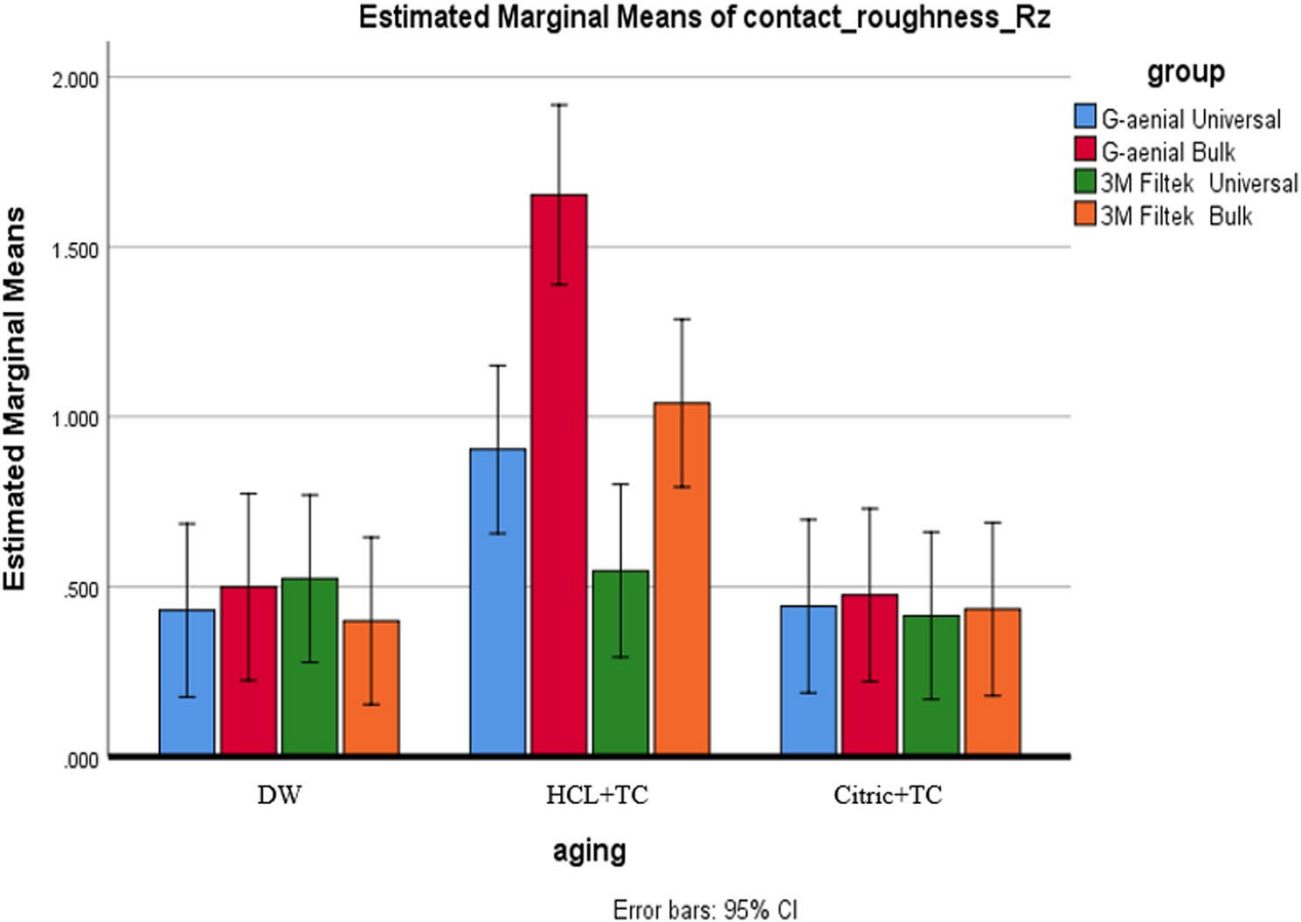
Comparison of Rz values among the different types of composites under each aging condition

In the DW condition, no significant differences in roughness were found between composites (Ra: *p* = 0.445; Rz: *p* = 0.889), with Ra values ranging from 0.061 μm to 0.108 μm and 95% CIs falling between [0.015–0.175], as shown in Figs. 2 and 3. After exposure to HCL + TC, G-aenial Bulk exhibited significantly higher roughness (Ra = 0.443 μm, 95% CI [0.279–0.568]; Rz = 1.653 μm, 95% CI [1.448–1.858]) than all other composites (*p* < 0.001), as indicated by superscript B. In contrast, G-aenial Universal (Ra = 0.152 μm, 95% CI [0.097–0.207]) and 3 M Filtek Bulk (Ra = 0.163 μm, 95% CI [0.050–0.276]) shared superscript A, indicating no significant difference. With Citric + TC, the differences between the composites were not statistically significant (Ra: *p* = 0.771; Rz: *p* = 0.989), but the roughness levels were ranked as follows: G-aenial Bulk (Ra = 0.088 μm, 95% CI [0.075–0.101]) was the roughest, followed by G-aenial Universal (Ra = 0.082 μm, CI [0.030–0.134]), then 3 M Filtek Bulk (Ra = 0.062 μm, CI [0.011–0.113]), and finally 3 M Filtek Universal (Ra = 0.063 μm, CI [0.023–0.103]).

#### Aging condition effect

**Fig. 4:**
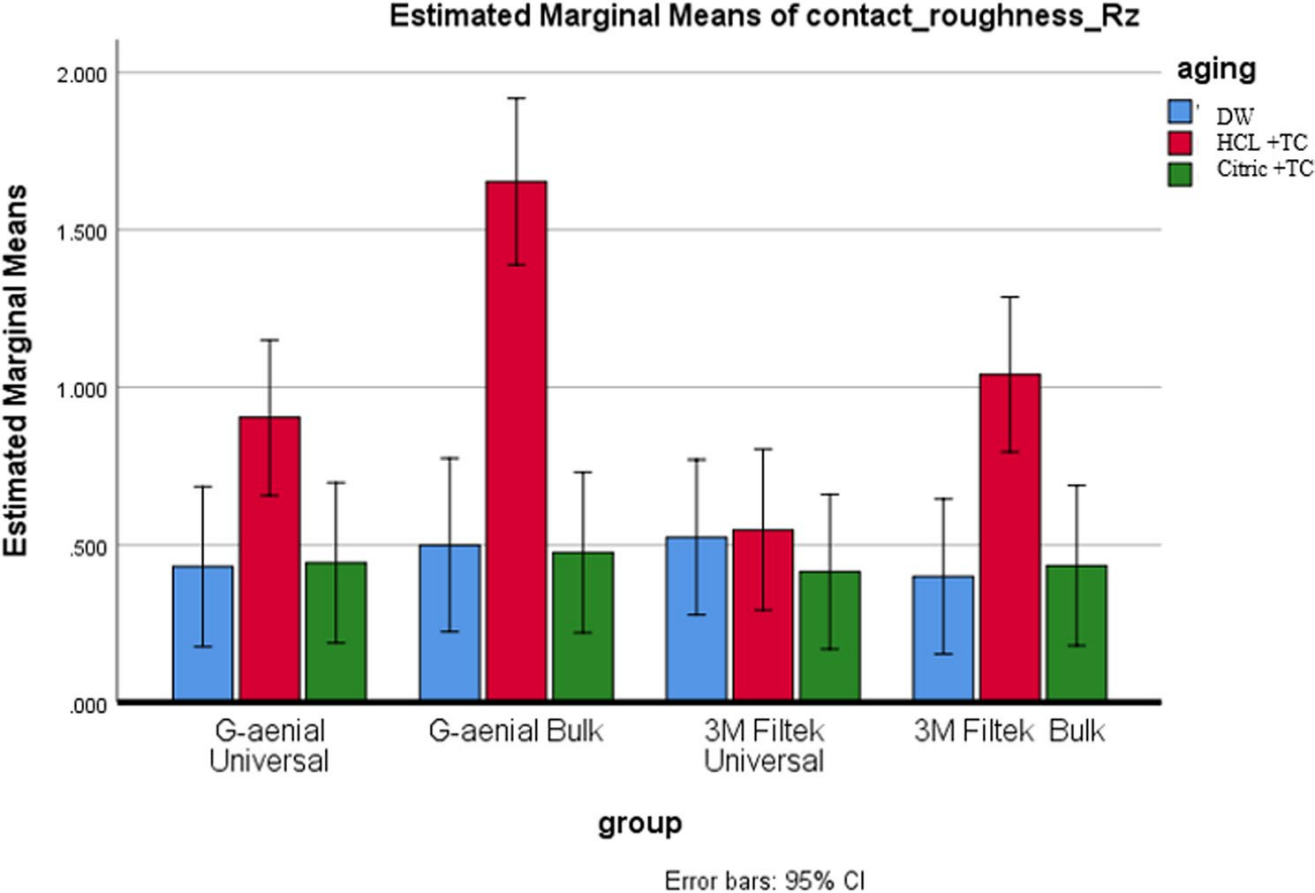
Provides a comparison of Ra across different aging conditions for each type of composite

**Fig. 5:**
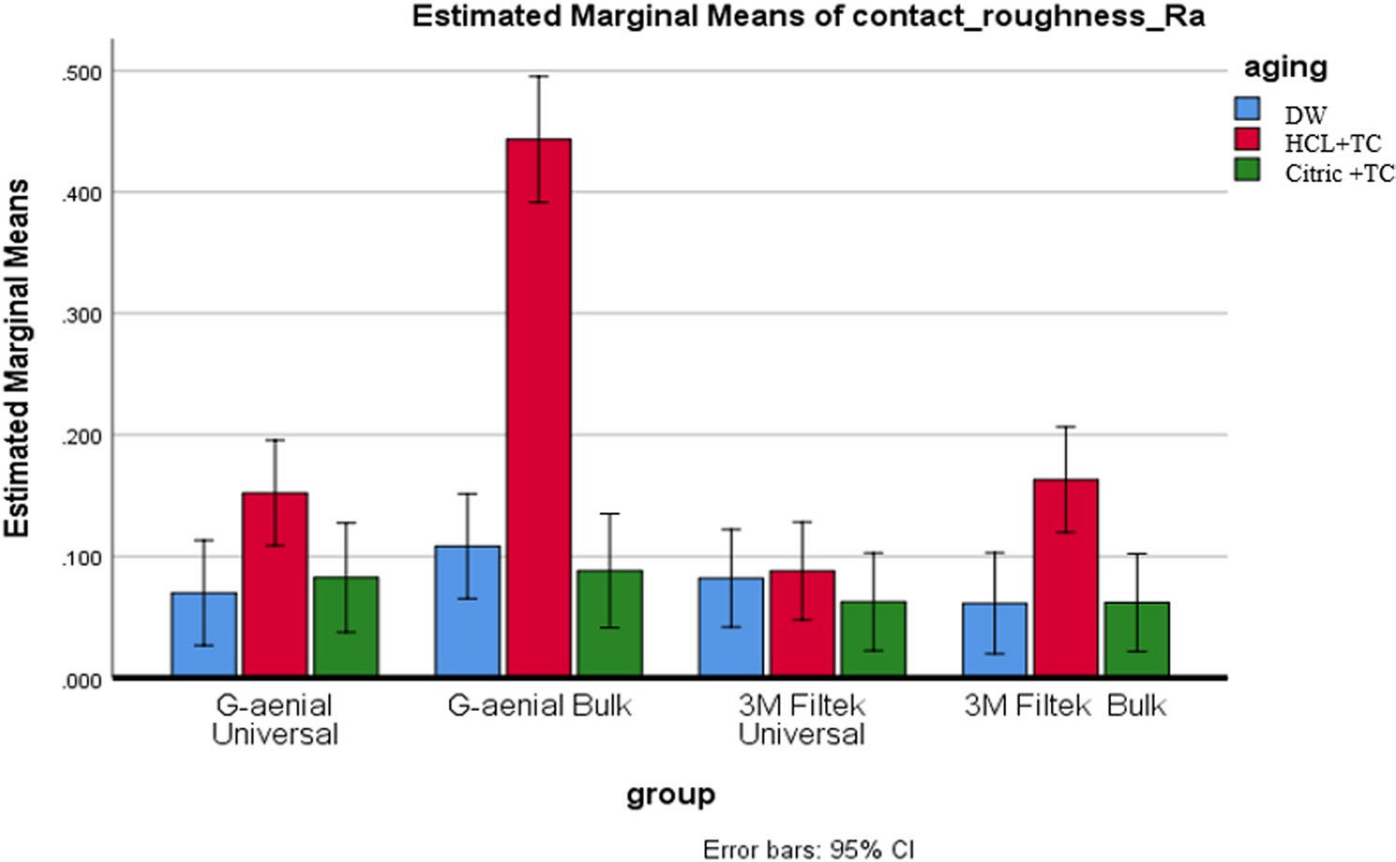
Compares the Rz values for each type of composite across various aging conditions

All composites showed the highest roughness after HCL + TC, followed by Citric + TC, with the lowest values in DW (*p* < 0.001). G-aenial Bulk demonstrated significant differences across all three conditions (*p* < 0.05). For the remaining composites, both Citric + TC and HCL + TC resulted in significantly greater roughness compared to DW (*p* < 0.05), but they did not show a significant difference from each other (*p* > 0.05), as illustrated in Figs. 4 and 5.

### Roughness results by AFM

**Fig. 6:**
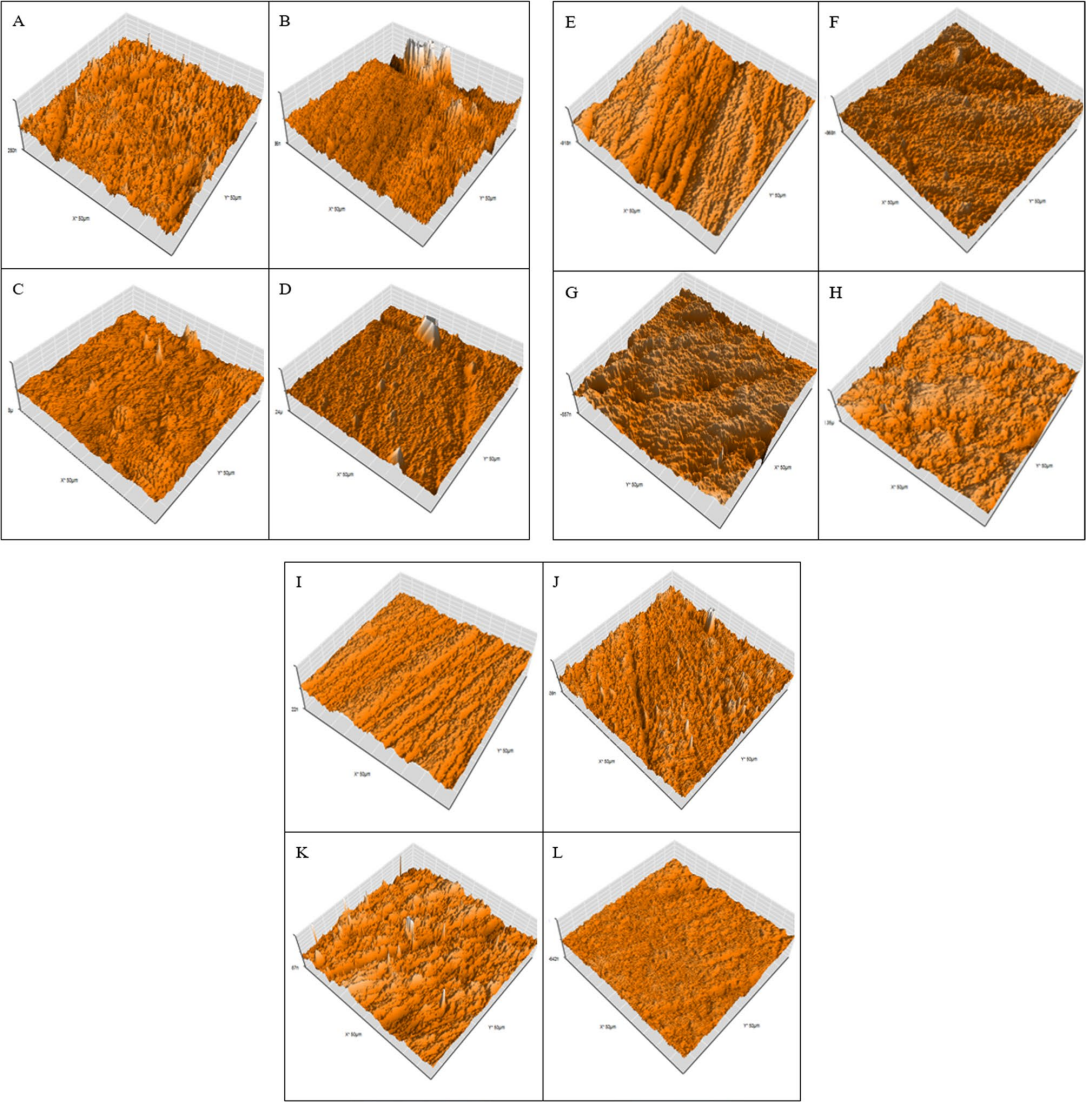
presents a 3D image that illustrates the representative surface roughness measured using AFM at a magnification of ×120. The following materials from the DW subgroup are represented: (**A**) for the G-aenial Universal, (**B**) for the G-aenial Bulk, (**C**) for the 3 M Filtek Universal, and (**D**) for the 3 M Filtek Bulk. The image features a selection of resin composites from the HCL + TC subgroup, which includes the G-aenial Universal, the G-aenial Bulk, the 3 M Filtek Universal, and the 3 M Filtek Bulk. These composites are labeled as (**E**), (**F**), (**G**), and (**H**), respectively. In the Citric + TC subgroup, the resin composites are labeled (**I**), (**J**), (**K**), and (**L**). Specifically, (**I**) refer to the G-aenial Universal; (**J**) denotes the G-aenial Bulk, (**K**) represents the 3 M Filtek Universal, and (**L**) indicates the 3 M Filtek Bulk

Table 5; Fig. 6 summarize AFM analysis across aging conditions, accompanied by representative 3D surface images at approximately ×120 magnification.

**Table 5:** Results of surface roughness measured by AFM (in nanometers)

Specimen	DW		HCL + TC		Citric + TC	
	Ra	Rz	Ra	Rz	Ra	Rz
G-aenial Universal	27.842	192.8	88.608	698.84	46.381	259.23
G-aenial Bulk	41.696	310.24	33.584	284.91	33.138	268.21
3 M Filtek Universal	43.678	298.68	35.678	207.91	31.848	263.88
3 M Filtek Bulk	39.623	302.61	94.066	595.55	36.754	220.01

#### Composite comparison

Deionized Water (DW): 3 M Filtek Universal showed the highest Ra, followed by G-aenial Bulk, 3 M Filtek Bulk, and the lowest in G-aenial Universal. Rz was highest in G-aenial Bulk and lowest in G-aenial Universal. HCL + Thermal Cycling (HCL + TC): 3 M Filtek Bulk had the highest Ra; Rz peaked in G-aenial Universal. G-aenial Bulk exhibited the lowest Ra under this condition. Citric Acid + Citric + TC: Ra was highest in G-aenial Universal; Rz was highest in G-aenial Bulk.

#### Aging condition effects

G-aenial Universal: Ra and Rz increased most in HCL + TC, followed by Citric + TC, and were lowest in DW. G-aenial Bulk: DW resulted in the highest roughness, followed by HCL + TC; Citric + TC showed the lowest surface roughness outcome. 3 M Filtek Universal: The Highest roughness was observed in DW, with lower values in HCL + TC and Citric + TC. 3 M Filtek Bulk: HCL + TC led to the highest Ra and Rz, followed by DW, with Citric + TC producing the lowest roughness.

#### SEM evaluation

**Fig. 7:**
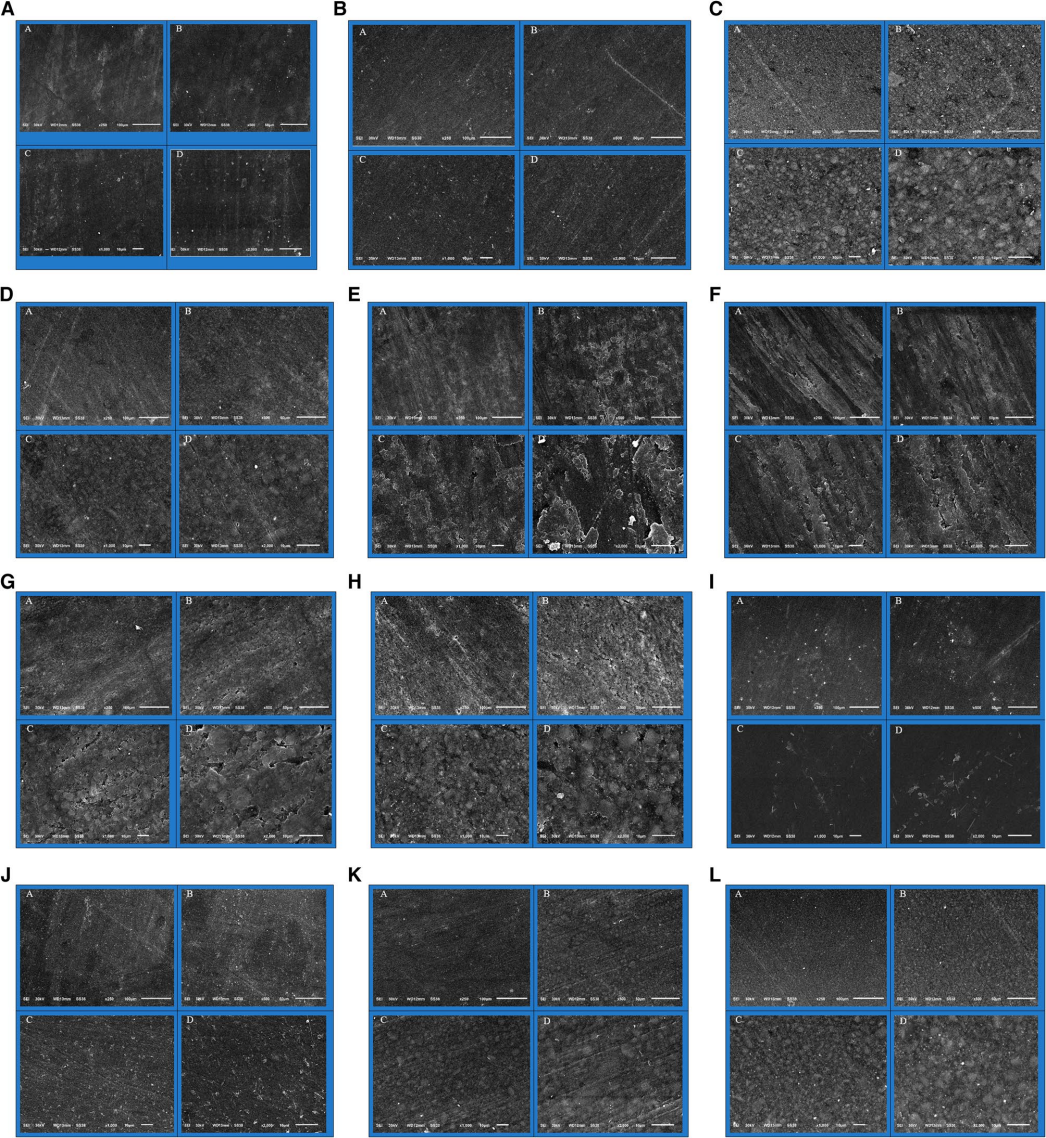
Displays a scanning electron microscope image of G-aenial Universal specimens from the DW subgroup. The surfaces of these representative specimens, examined at magnifications of ×250 and ×500 (labeled as **A** and **B**), show a low degree of roughness with few striations. Visual inspection reveals uniform filler particles embedded within the resin matrix, with no discernible striations at higher magnifications of ×1000 and ×2000 (**C** and **D**). Presents the micrographs of the G-aenial Bulk from the DW subgroup. Composite disc micrographs were captured at magnifications of ×250 (**A**) and ×500 (**B**). Visual inspection indicated the presence of homogeneous filler particles encapsulated within a resin matrix, exhibiting minimal to no striations. At higher magnifications of ×1000 and ×2000 (**C**, **D**), faint striations became noticeable. Presents micrographs of the 3 M Filtek Universal from the DW subgroup. At a magnification of ×250 (**A**), the surfaces exhibit striations and uniformly distributed filler particles embedded in a resin matrix. At higher magnifications of ×500, ×1000, and ×2000 (**B**, **C**, **D**), faint striations remain visible, suggesting low and consistent surface roughness. Presents micrographs of the 3 M Filtek Bulk from the DW subgroup. The surface of the sample showed Lines and evenly distributed filler particles mixed in with the resin. The composite disc micrographs were captured at magnifications of approximately 250 and 500 (**A**, **B**). Further examination of the specimen was conducted at magnifications of approximately 1000 and 2000 (**C**, **D**). Additionally, the presence of scratches and surface roughness related to the polishing procedure was noted. Shows micrographs of the G-aenial Universal from the HCL + TC subgroup; striations are visible on the composite discs after both HCL exposure and thermal cycling at a magnification of ×250 (**A**), indicating small micro-retentive fissures and significant surface roughness. At magnifications of ×500, ×1000, and ×2000 (**B**, **C**, **D**), the surfaces also exhibited substantially deeper pitting and irregular porosities due to filler leaching, creating the appearance of a rougher surface across the field of view. Shows the micrographs of the G-aenial Bulk from the HCL + TC subgroup. At each magnification (×250, ×500, ×1000, and ×2000), the surfaces displayed a relatively rough, striated texture with deep micro-retentive fissures. The filler appeared to be exposed or degraded due to acidic exposure and thermal cycling, resulting in a highly irregular surface characterized by deeper pitting and irregular porosities. Presents the micrographs of the 3 M Filtek Universal from the HCL + TC subgroup. Across the field of view, the surfaces at magnifications of ×250, ×500, and ×1000 (**A**, **B**, and **C**) appeared rough and uneven, showing deep pitting and irregular porosities. At magnification ×2000 (**D**), the resin matrix exhibited further deterioration, leading to additional surface pitting. Shows the micrographs of the 3 M Filtek Bulk for the HCL + TC subgroup. At magnifications of ×250 (**A**) and ×500, ×1000, and ×2000 (**B**, **C**, and **D**), the surfaces appear rough and uneven, exhibiting significantly deeper pitting and irregular porosities. The higher magnifications also reveal the deterioration of the resin matrix throughout the field of view. Displays the micrographs of the G-aenial Universal for the Citric + TC subgroup. At magnifications of ×250 and ×500 (**A**, **B**), the surfaces appear somewhat rougher, yet they are comparable to those of the DW subgroup. In contrast, at magnifications (**C**, **D**), the surfaces are significantly smoother than those in the HCL + TC subgroup. Additionally, mild filler exposure is observed throughout the field of view. Shows the micrographs of the G-aenial Bulk from the Citric + TC subgroup. It illustrates the breakdown of the resin matrix and the exposure of filler particles, which appear relatively rough on the surface. Qualitative changes are observed in the surfaces of the composite discs at each magnification of ×250, ×500, ×1000, and ×2000 (**A**, **B**, **C**, and **D**). Presents the micrographs of 3 M Filtek Universals from the Citric + TC subgroup, illustrating qualitative changes on the surfaces of composite discs A, B, C, and D at magnifications of ×250, ×500, ×1000, and ×2000. The striated appearance of the surfaces reflects the grit or coarseness used during application. Additionally, a slight roughness and exposure of the filler material are noted in discs B, C, and D at magnifications of ×500, ×1000, and ×2000. Shows the micrographs of the 3 M Filtek Bulk from the Citric + TC subgroup. The micrographs reveal rough surfaces, indicating qualitative variations in the composite disc surfaces at magnifications of ×250 and ×500 (**A** and **B**). In contrast, at magnifications of ×1000 and ×2000 (**C** and **D**), mild surface roughness with filler exposure is observed, attributed to the degradation of the resin matrix

Figure 7 shows the results of the SEM investigation for G-aenial Universal, G-aenial Bulk, 3 M Filtek Universal, and 3 M Filtek Bulk.

### Vickers microhardness

A two-way ANOVA on Vickers microhardness values showed Composite type effect: significant (*p* < 0.001), Aging condition effect: not significant (*p* = 0.323), Composite × Aging interaction: significant (*p* = 0.031), The result indicates that aging impacts microhardness differently depending on the composite type, so post-hoc comparisons were performed within each aging subgroup. See Table 6.

**Table 6:** Shows the Vickers microhardness values for all the composite groups tested under different aging conditions, including several comparisons, averages, standard deviations, and p-values

	DW	HCL + TC	Citric + TC	DW-HCL + TC	DW-Citric + TC	HCL + TC-Citric + TC
g/µm²	**Mean**,** (SD)**	**Mean**,** (SD)**	**(Mean**,** SD)**			
G-aenial Universal	70.49^A^, **(**1.12)	65.72^A^, (0.95)	66.43^A^, (0.82)	< 0.001*	< 0.001*	0.117
G-aenial Bulk	68.91^B^, (0.79)	64.59^B^, (1.87)	66.64^A^, (1.00)	< 0.001*	< 0.001*	< 0.001*
3 M Filtek Universal	68.52^B^, (1.21)	65.71^A^, (0.88)	65.83^A^, (0.79)	< 0.001*	< 0.001*	0.795
3 M Filtek Bulk	68.88^B^, (0.64)	65.69^A^, (0.67)	66.36^A^, (0.73)	< 0.001*	< 0.001*	0.140

p* is significant at the 5% level. Distinct letters inside the same column indicated a statistically significant difference between each pair of groups (Bonferroni test, p < 0.05). Identical letters inside the same column indicated no statistically significant difference between the two groups (Bonferroni test, p > 0.05) in all following tables

*X* mean, *SD* standard deviation

#### Effect of composite type

**Fig. 8:**
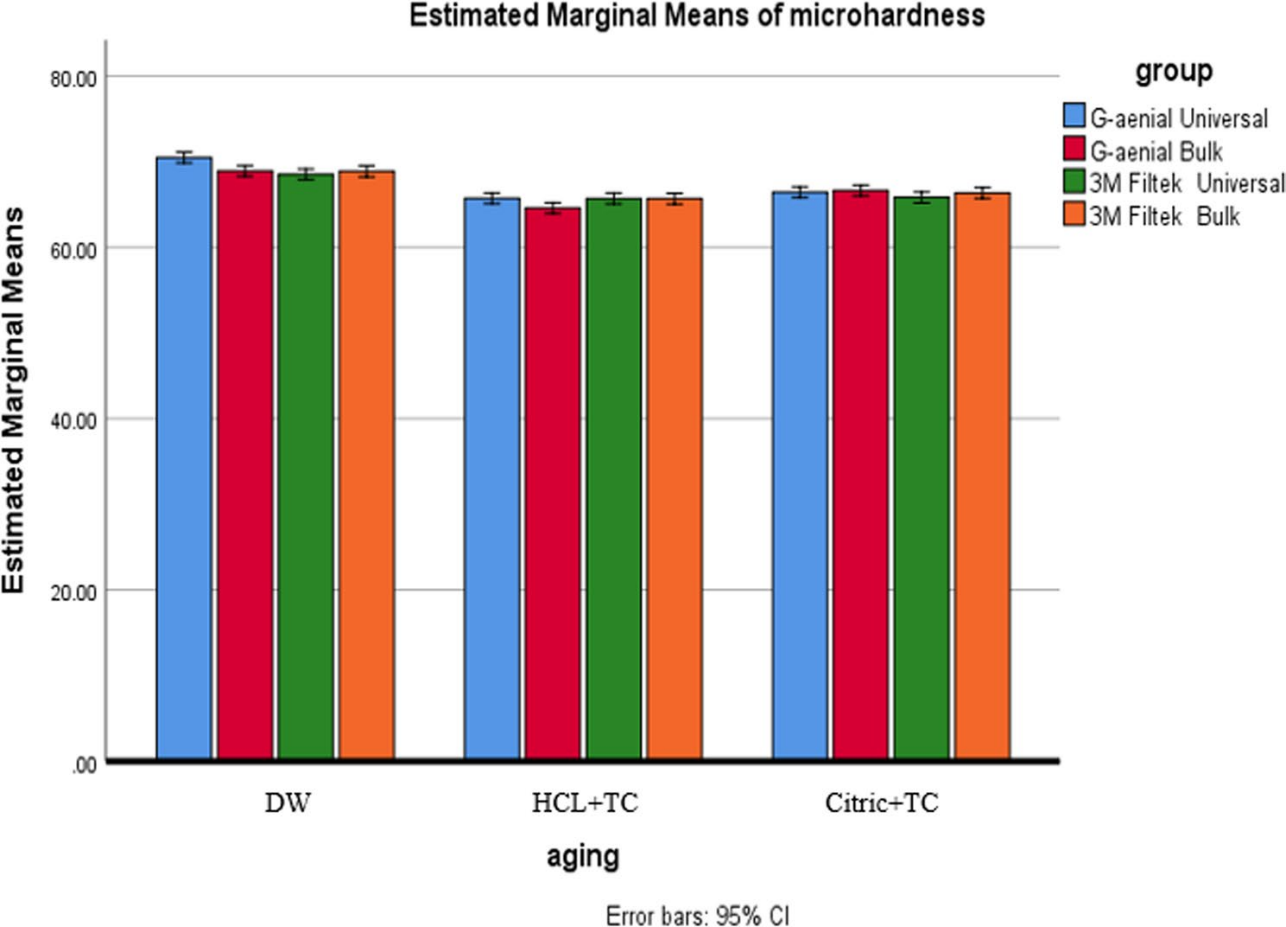
Comparison of microhardness across different composite types under various aging conditions

In DW, G-aenial Universal had significantly higher hardness (e.g., 80.2 HV, 95% CI [77.1–83.3]) than the other composites (*p* < 0.001). The other three—G-aenial Bulk, 3 M Filtek Bulk, and 3 M Filtek Universal—had overlapping CIs, indicating no significant differences. After HCL + TC (Acid + Thermal Cycling): G-aenial Bulk had the lowest hardness (61.4 HV, 95% CI [58.2–64.6]), significantly lower than others (*p* = 0.031). 3 M Filtek Universal remained the hardest under this condition, with its CI not overlapping that of G-aenial Bulk. Under Citric + TC: No significant differences were detected—all CIs overlapped (e.g., G-aenial Bulk 65.1 HV [62.0–68.2]; G-aenial Universal 64.3 HV [61.1–67.5]). See Fig. 8.

#### Effect of aging condition

**Fig. 9:**
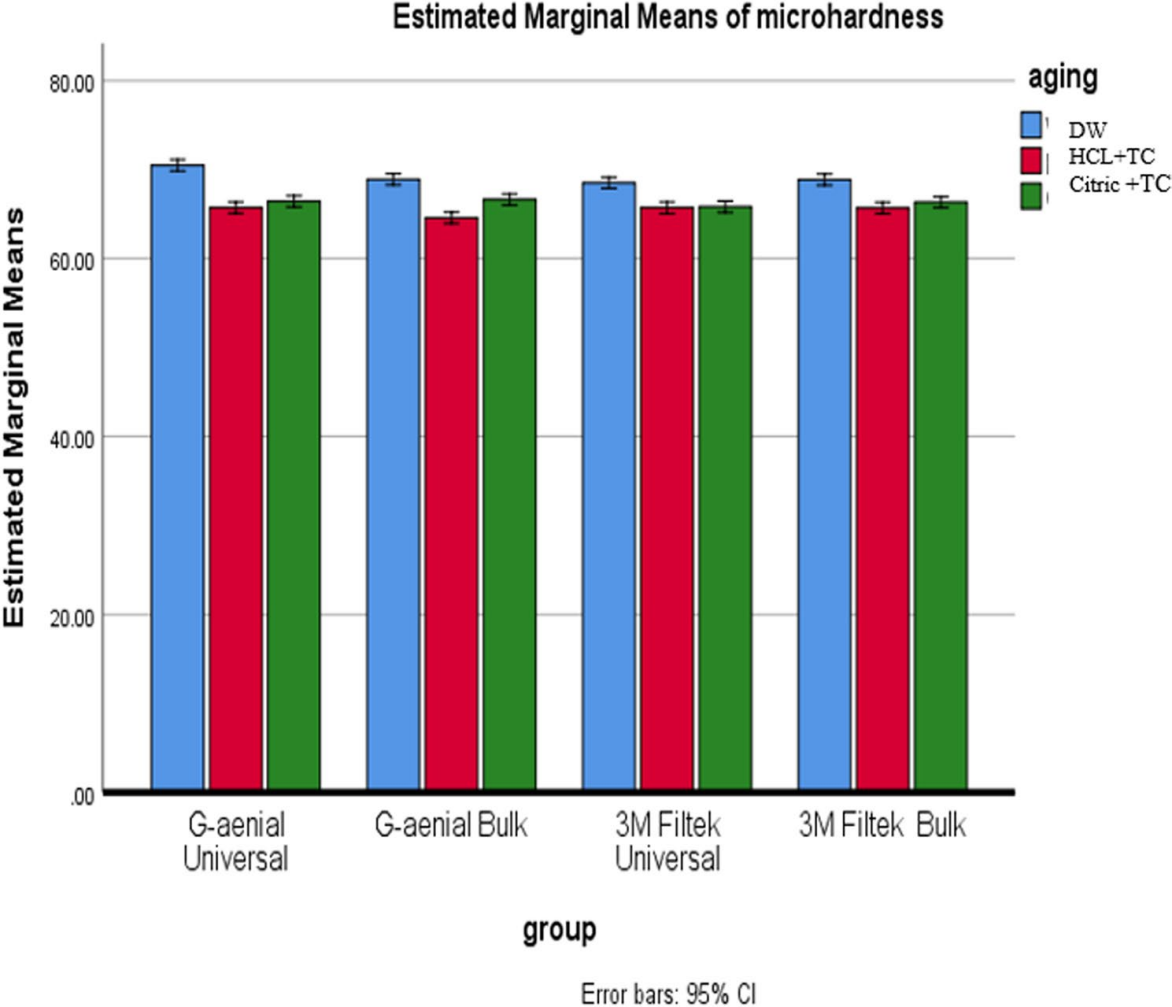
Comparison of microhardness under various aging conditions for each type of composite

Across all composites, hardness ranked as DW > Citric + TC > HCL + TC (*p* < 0.001). G-aenial Bulk showed statistically significant differences across all three conditions (non-overlapping CIs). For the other composites (G-aenial Universal, 3 M Filtek Universal, 3 M Filtek Bulk), both acid-treated conditions were significantly lower than DW (*p* < 0.05), but HCL + TC and Citric + TC showed similar results (CIs overlapped). See Fig. 9.

### Wear by weight loss

A two-way ANOVA revealed a significant interaction (*p* = 0.003), prompting simple post-hoc analyses using the Bonferroni correction. See Table 7.

**Table 7:** Comparison of wear measured by weight loss (in grams) between composite types and aging circumstances, encompassing averages, standard deviations, and p-values

	DW	HCL + TC	Citric + TC
	**Mean**,** (SD)**	**Mean**,** (SD)**	**Mean**,** (SD)**
G-aenial Universal	−0.0028^A^, (0.0022)	−0.0065^A^, (0.0049)	−0.0019^A^, (0.0020)
G-aenial Bulk	−0.0025^A^, (0.0023)	−0.0087^A^, (0.0068)	−0.0531^B^, (0.0923)
3 M Filtek Universal	−0.0078^A^, (0.0030)	−0.0132^A^, (0.0077)	−0.0067^A^, (0.0042)
3 M Filtek Bulk	−0.0023^A^, (0.0005)	−0.0113^A^, (0.0042)	−0.0022^A^, (0.0007)

p* is significant at the 5% level. Distinct letters inside the same column indicated a statistically significant difference between each pair of groups (Bonferroni test, p < 0.05). Identical letters inside the same column indicated no statistically significant difference between the two groups (Bonferroni test, p > 0.05) in all following tables

*X* mean, *SD* standard deviation

#### Effect of the composite type

**Fig. 10:**
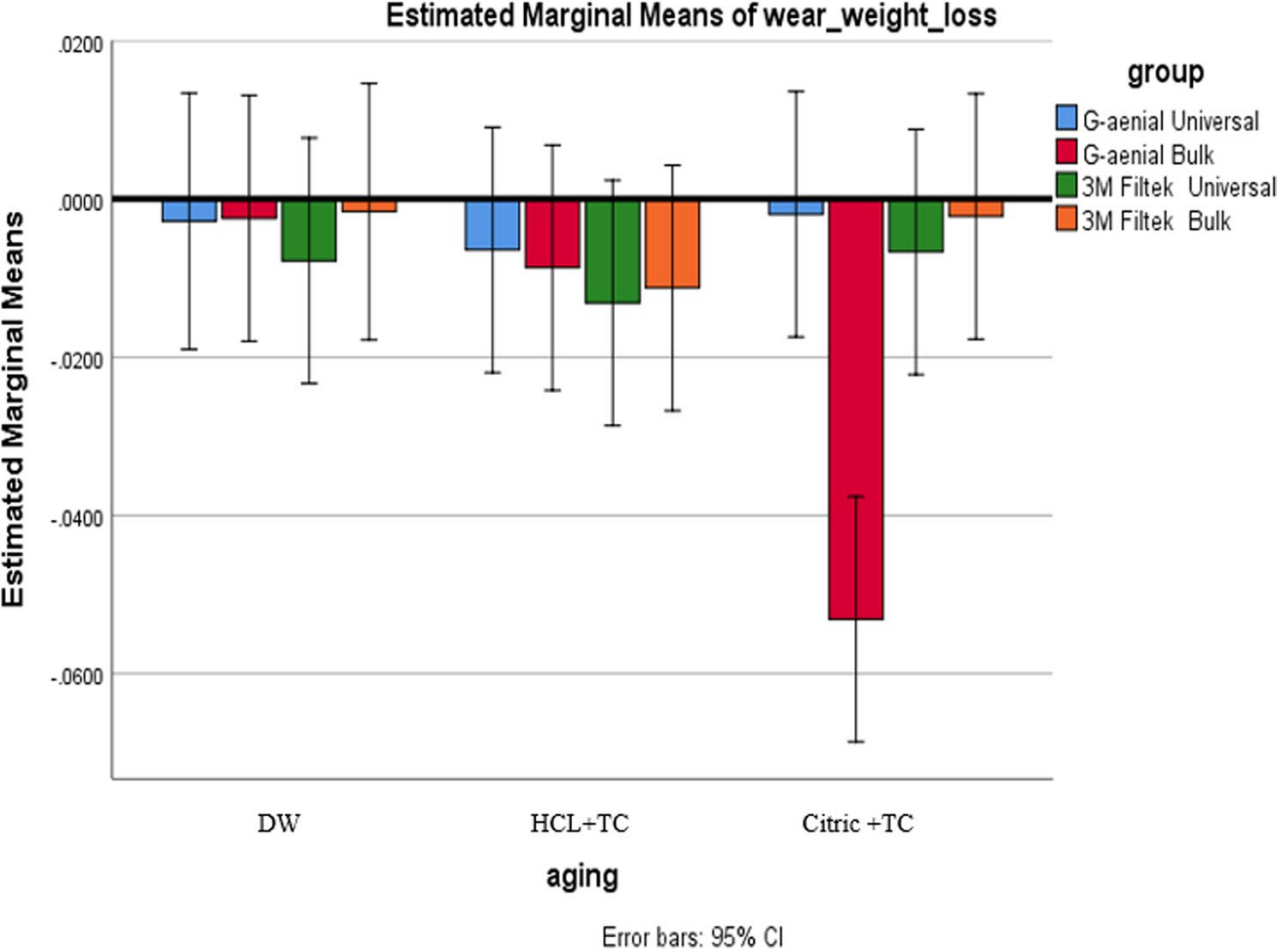
Compares wear, measured by weight loss, among different composite types for each aging condition

Under Citric + TC, G-aenial Bulk exhibited significantly higher weight loss than all other composites (Bonferroni-adjusted 99.2% CIs excluded zero; *p* < 0.01). Under DW and HCL + TC, no pairwise compositional differences were significant. This confirms that while composite type matters under Citric + TC, there are no meaningful overall aging-condition effects (*p* = 0.093) or generalized differences across all composites as shown in Fig. 10.

#### Effect of aging conditions (within each composite)

**Fig. 11:**
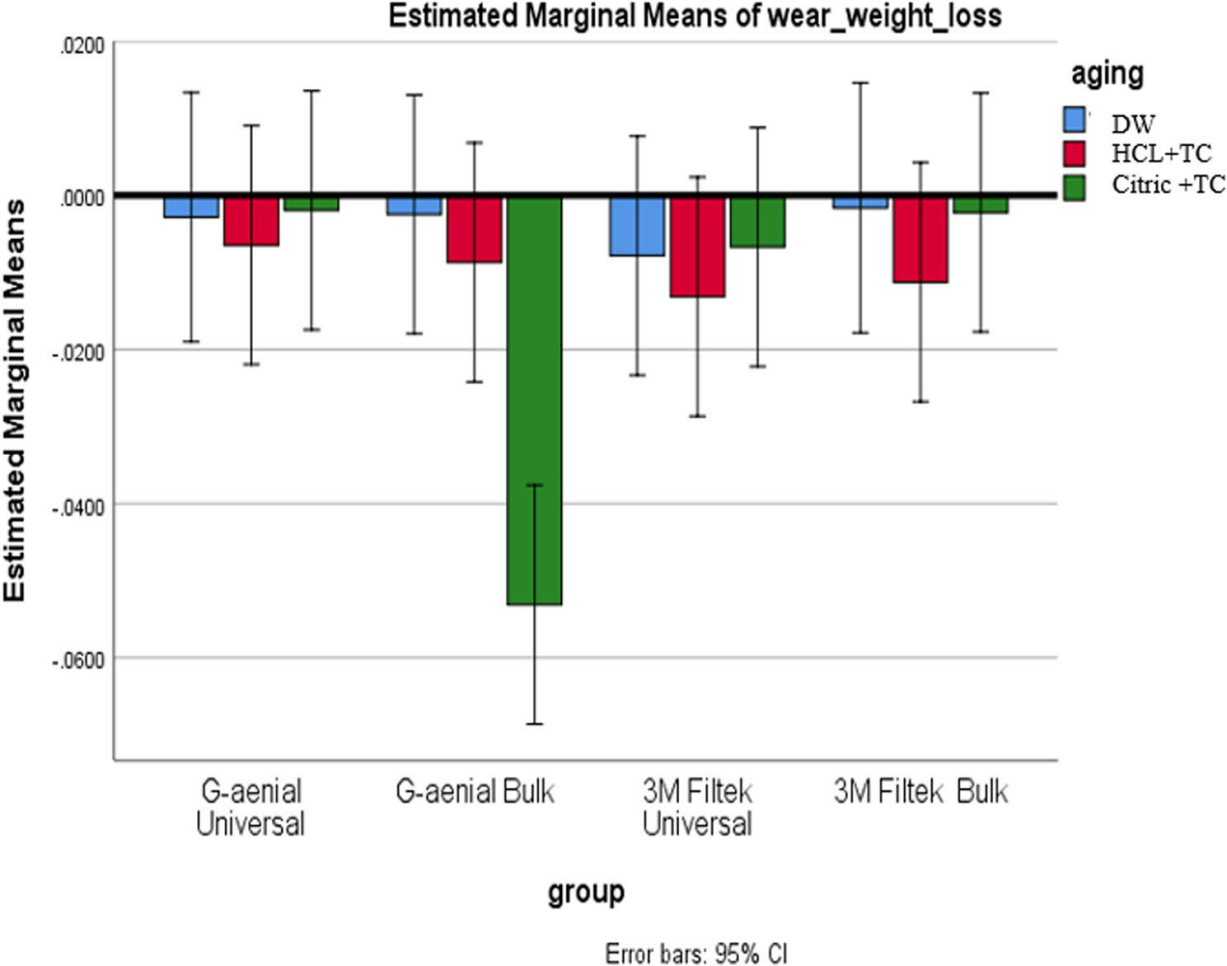
Shows a comparison of wear due to weight loss across different aging conditions for each type of composite

Across all composites, weight loss did not significantly differ between aging conditions (*p* > 0.05). Despite non-significance, the observed trend was HCL + TC > DW > Citric + TC in terms of average wear for most materials as shown in Fig. 11.

### Wear by volume loss

**Fig. 12:**
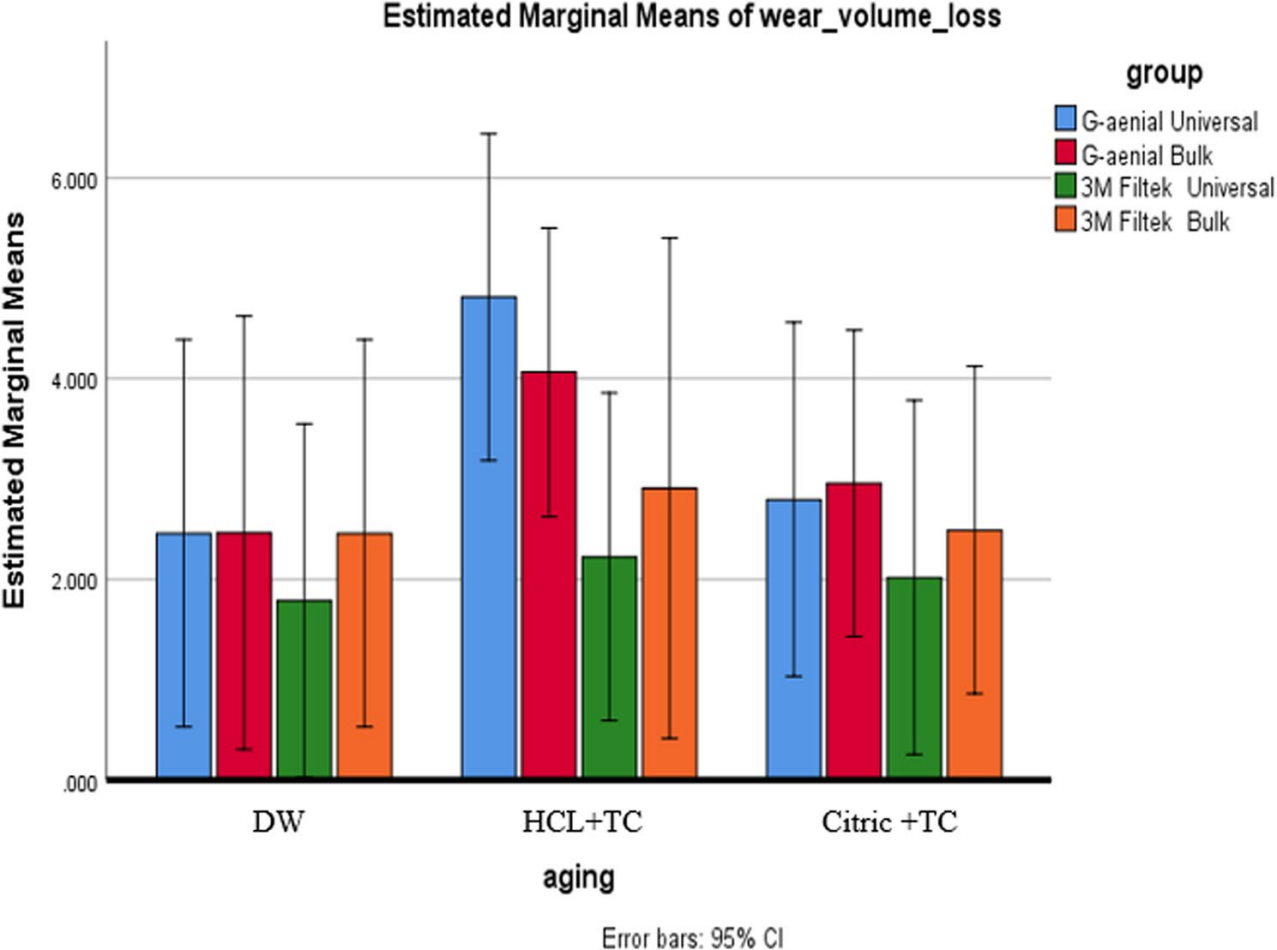
Displays a comparison of wear measured by volume loss among different types of composites under various aging conditions

**Fig. 13:**
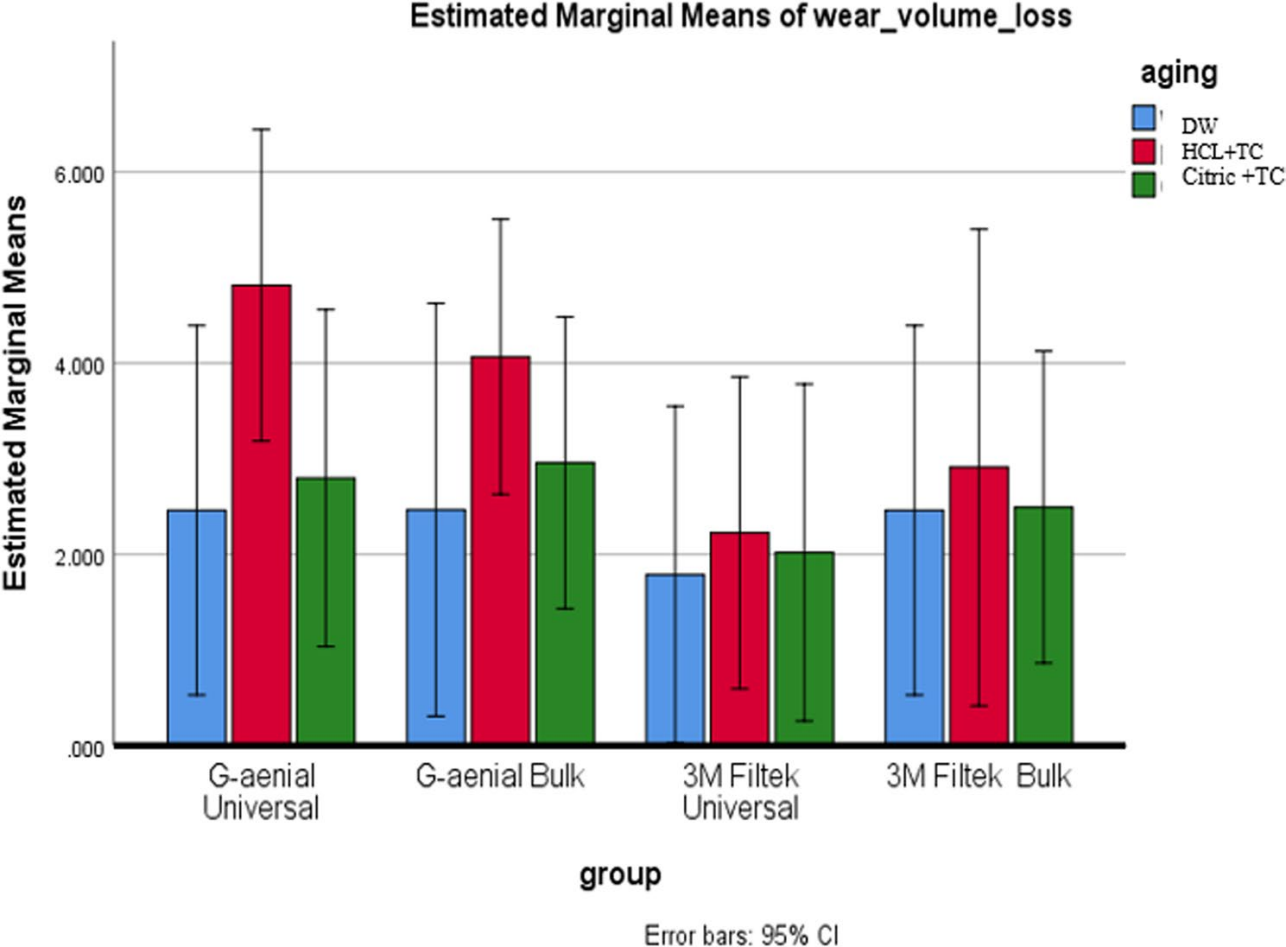
Compares the wear characteristics based on volume loss under different aging conditions for each composite type

A two-way ANOVA showed no significant effects of composite type (*p* = 0.240), aging condition (*p* = 0.165), or their interaction (*p* = 0.919) on volume loss. Post-hoc 95% confidence intervals for pairwise comparisons mostly included zero—indicating no statistically meaningful differences (except in a few cases such as HCL vs. DW for G-aenial Universal and Bulk). These CI results fully support the ANOVA’s conclusion: overall mean volume losses do not differ significantly across composites or aging conditions. As shown in Table 8; Figs. 12 and 13, mean volume loss values are similar across both composites and aging conditions.

**Table 8:** Comparison of wear by volume loss among composite types and aging conditions, including means, standard deviations, and p-values

	DW	HCL + TC	Citric + TC
(µm³)	**Mean**,** (SD)**	**Mean**,** (SD)**	**Mean**,** (SD)**
G-aenial Universal	2.47^A^, (2.64)	4.81^A^, (2.23)	2.80^A^, (1.81)
G-aenial Bulk	2.46^A^, (2.16)	4.07^A^, (2.48)	2.96^A^, (2.72)
3 M Filtek Universal	1.79^A^, (0.97)	2.22^A^, (1.70)	2.02^A^, (1.64)
3 M Filtek Bulk	2.46^A^, (2.64)	2.91^A^, (2.48)	2.49^A^, (1.81)

p* is significant at the 5% level. Distinct letters inside the same column indicated a statistically significant difference between each pair of groups (Bonferroni test, p < 0.05). Identical letters inside the same column indicated no statistically significant difference between the two groups (Bonferroni test, p > 0.05) in all following tables

*X* mean, *SD* standard deviation

## Discussion

Regardless of the aging process, the study’s results revealed significant differences in the roughness, microhardness, and wear resistance of the investigated resin composites. As a result, the first null hypothesis was rejected, while the second null hypothesis was partially accepted. Aging conditions affected the roughness and microhardness of the tested resin composites but had no impact on their wear resistance. Study findings align with Degirmenci et al. (2022) [45] who reported that low-pH exposure markedly increases surface roughness and reduces microhardness in injectable composites after immersion in acidic beverages like orange juice. Similarly, Svizero et al. (2014) [46] found that the hardness of nanofilled composites gradually decreased when exposed to acidic environments. Gil-Pozo and Medina-Sotomayor (2022) [47] showed that simulated gastric acid greatly weakens the hardness and bending strength of regular composites, emphasizing how natural acids can make materials softer. Conversely, Alencar et al. (2020) [48] found that bulkfill and nanofilled composites maintain smooth surfaces and hardness when challenged by acidic conditions, suggesting these materials’ resilience in clinical settings. Wang et al. (2013) [49] also reported that reducing filler size and enhancing matrix bonding via salinization technologies improves composite integrity under stress. Finally, Guo et al. (2022) [9] indicated a direct correlation between surface roughness and wear in citric acid environments, underscoring roughness as a predictor of material degradation.

In this study, the stylus profilometer was used to assess roughness. Within the HCL + TC subgroup, a significant difference in surface roughness (Ra, Rz) was observed between the types of composites, except between G-aenial Universal and 3 M Filtek Bulk. The highest Ra and Rz values were recorded for G-aenial Bulk, followed by 3 M Filtek Bulk, then G-aenial Universal, with the lowest Ra and Rz observed for 3 M Filtek Universal. Current study findings suggest that bulkfill composites are more prone to water-induced degradation than traditional composites, allowing deeper penetration of water and acids and leading to hydrolytic breakdown, softening, and swelling of the resin matrix [50]. The organic matrix is the weakest part of the composite, and it’s likely that its breakdown from water and chemicals on the surface caused it to lose its roughness and hardness. The matrix exhibits high sensitivity to water absorption [50, 51]. Additionally, the differing coefficients of thermal expansion between the polymer matrix and filler particles likely induced interfacial stresses during thermal cycling, further weakening resin–filler bonds [52]. Moreover, all composites, with the exception of G-aenial Bulk, utilize silica as a filler. G-aenial Bulk exclusively utilizes barium glass as its filler material, whereas G-aenial Universal incorporates barium glass as well. The pH of the surrounding environment can substantially affect the degradation of various filler materials. Filler particles, such as silica and barium glass, may deteriorate when the pH decreases. Barium glass can break down more easily when it comes into contact with water due to its positive charge, which may lead to a weakening of the bond between the filler and the polymer. Furthermore, surface roughness escalates as barium glass is more readily soluble in acidic solutions compared to silica [53]. This mechanism explains the more pronounced roughening observed in G-aenial Bulk versus others under erosive aging conditions.

The dispersed nano-sized barium particles (150 nm) in G-aenial Universal, which are firmly bonded to the resin matrix using full coverage silane coating (FSC) technology, may have contributed to a strong and stable filler-matrix bond [54]. Nanoparticles and nanocluster fillers make up 78.5% of the weight of 3 M Filtek Universal. The silica and zirconia fillers are separate, non-clumped nanoparticles that measure 20 nm and 4–11 nm in size, respectively. Furthermore, the nanocluster particles enhance the nanocomposite’s filler loading, physical properties, and polish retention. The smooth surfaces of 3 M Filtek Universal observed in the current study within the HCL + TC subgroup, compared to the other three tested composites, can be attributed to the uniform dispersion of precured silica particles within the organic matrix [55]. In agreement with the current study, Aydinoğlu et al. (2017) [56] reported that the surface roughness of G-aenial Universal was lower compared to other tested flowable composites. The low surface roughness of injectable resin composites may be attributed to better filler salinization, which enhances their physical properties.

Regarding the effect of aging conditions on composites, contact profilometer data for G-aenial Universal and 3 M Filtek Bulk revealed that the DW had the lowest roughness (Ra), followed by Citric + TC, and HCL + TC had the highest roughness. In the case of G-aenial Bulk and 3 M Filtek Universal, Citric + TC exhibited the lowest roughness, unlike the DW subgroup. Acids contain enough protonated ions (H+) to catalyze the hydrolysis of ester groups in the matrix. The products of this hydrolysis, such as alcohols and carboxylic compounds, exacerbate deterioration by lowering the matrix’s pH. Acids can also cause erosion on filler surfaces, leading to filler leaching and a rougher surface [47]. Moreover, hydrolytic breakdown of the bond between silane and the filler particles may result in filler-matrix debonding [57].

Chemically, HCL acts as a strong plasticizer that accelerates water sorption and solubility, promoting the dissolution of the resin composites [58]. Furthermore, the depth of surface roughness may be significantly influenced by the temperature at which acidic solutions are tested [15]. Due to the reciprocal action of the citric acid molecule’s hydrophilic carboxyl group, which may be adsorbed chemically or physically onto the material’s surface, combined with the increase in the microenvironment’s temperature, a combination of these effects may occur [9]. The leaching of filler particles weakens the resin composite when submerged in organic acids [59]. Additionally, the plasticizing effect of water and the hydrolysis of silane coupling agents can enhance water sorption in resin composites during thermal cycling, further degrading the material’s surface [17]. In the current study, the effect of citric acid was limited to the formation of a thin, single-layer or multilayer boundary lubricating layer on the specimen’s surface [59]. This layer prevents further acid penetration into the resin matrix, resulting in less degradation and roughness. This phenomenon could explain the lower roughness of the tested materials after exposure to citric acid compared to HCL. Eating fruits and drinking acidic liquids can create a transient citric acid environment in the oral cavity, which may wash away or wear down the boundary lubricant layer [9].

It has been observed that a solution’s erosive potential depends not only on its low pH but also on factors such as titratable acidity, acid concentration, type of acid, immersion period in acidic liquids, and beverage composition [60]. Citric acid can damage surfaces by binding to and moving between particles and acid ions, which shows how important these actions are when looking at how damaging a solution can be. Additionally, zirconia/silica fillers can break down in water when they come into contact with acid, and the smaller spherical zirconia/silica fillers that are attached to the resin matrix tend to dissolve more easily [59]. In contrast, 3 M Filtek Universal absorbed less water than bulkfill composites [50], even though it had zirconia/silica fillers, while 3 M Filtek Universal only had silica fillers. G-aenial Bulk, on the other hand, did not contain zirconia/silica fillers. These factors may help explain why citric acid had a lesser effect than DW on G-aenial Bulk and 3 M Filtek Universal.

The results of the standard AFM roughness test are consistent with those obtained from the contact profilometers for G-aenial Universal and 3 M Filtek Universal. The current study showed that G-aenial Universal had greater surface roughness than 3 M Filtek Universal when aged with HCL + TC. In contrast, Basheer R. et al. (2024) [40] found no significant difference in surface roughness between G-aenial Universal and 3 M Filtek Universal composites. This difference might be because the specimens in the current study were heated and cooled after being soaked, while the specimens in Basheer R. et al.‘s study were only heated and cooled without being exposed to the aging solution.

Regarding the influence of the composite type on microhardness, regardless of aging conditions, G-aenial Universal exhibited the highest microhardness in the DW subgroup, while 3 M Filtek Universal had the lowest. The small barium particles (150 nm) in G-aenial Universal stick well to the resin because of FSC technology, which probably helps form a strong connection between the filler and the matrix, resulting in G-aenial Universal having a much higher VHN [33]. For the HCL + TC subgroup, the lowest microhardness was observed with G-aenial Bulk, while the highest was noted with 3 M Filtek Universal. This variation could be attributed to the higher filler loading of 3 M Filtek Universal (78.5% wt.) compared to G-aenial Bulk (69% wt.). Furthermore, bulkfill composites are more prone to water sorption and degradation than conventional types, which might explain this difference [50]. In terms of filler content, all composites except G-aenial Bulk contain silica, with G-aenial Universal also including barium glass, while G-aenial Bulk contains only barium glass. The pH of the surrounding environment can significantly affect the degradation of these filler materials. As pH decreases, filler particles like silica and barium glass become more susceptible to degradation. Specifically, barium glass is more likely to break down when it comes into contact with water because it has a positive charge, which can weaken the strength of the composite. Additionally, barium glass dissolves more readily in acidic solutions than silica, leading to a loss in surface microhardness [53]. The current study found that G-aenial Universal was harder on the surface in the DW subgroup compared to 3 M Filtek Universal, which goes against the findings of Basheer R. et al. (2024). This difference might be because the current study looked at the DW subgroup, which did not include thermal cycling. This discrepancy might be due to the current study focusing on the DW subgroup, which did not include thermal cycling [40]. The results of the current study pertained to the DW subgroup, which did not undergo thermal cycling; this may explain the observed discrepancy.

Regarding the effect of aging conditions on the VHN of resin composites, significant differences were observed among all composites except for G-aenial Bulk. The VHN values showed significant differences across aging conditions, except between HCL + TC and Citric + TC. G-aenial Bulk displayed significant variations in microhardness depending on the aging condition. DW resulted in the highest Vickers microhardness, followed by Citric + TC, while HCL + TC exhibited the lowest microhardness values. Resin composites are made up of different materials, usually including a plastic-like base, strengthening particles, silane, and other chemicals. Differences in the resin matrix and filler particles can lead to variations in surface properties, such as microhardness [61]. The durability of these materials is particularly influenced by their resistance to matrix disintegration and water absorption, both of which can be affected by acidic media [62]. These acids can dissolve the resin matrix, softening components like Bis-GMA and increasing the likelihood of unreacted monomers being released. Monomers such as UDMA, TEGDMA, and Bis-GMA are highly soluble and can contribute to the degradation and softening of the resin matrix [59].

In this study, weight loss and volume loss were used to assess wear. When looking at how different types of composites resist wear, regardless of aging conditions, there was a notable difference in wear based on weight loss for the Citric + TC aging condition. G-aenial Bulk exhibited the highest wear rate, while G-aenial Universal showed the lowest. A significant difference was found between G-aenial Bulk and all other composites, though no significant differences were detected among the remaining composites.

G-aenial Universal contains uniform spherical fillers with a diameter of 0.15 μm (150 nm). Historically, average filler volume and size have been associated with improved resin composite wear characteristics [63]. In resin composites, the size of the filler particles affects surface roughness and friction coefficient, which in turn influences wear resistance. Smaller particles improve wear resistance in areas without contact by bringing the filler particles closer together and lowering the internal stress in the polymer. With more closely spaced filler particles, the resin is better shielded from additional erosion by surrounding particles [64, 65]. Therefore, increasing filler loading and decreasing filler particle size could enhance wear resistance [24].

According to the current results, specimens of G-aenial Bulk showed the highest weight loss after attrition wear. This can be attributed to the direct localized contact between the specimens and the opposing tooth’s cusp tip during attrition, which causes localized microfractures at the relevant location [66]. One possible explanation is that bulkfill composites like G-aenial Bulk and 3 M Filtek Bulk are more susceptible to water-induced degradation than conventional composites [50]. The higher wear of G-aenial Bulk compared to other composites may be attributed to the absence of silica, as the improvement in mechanical properties resulting from the incorporation of silica nanoparticles applies to all composites except G-aenial Bulk [67].

Ujiie M. et al. (2020) [65] proposed that localized wear depends on the material makeup, noting that big clumps, like those in the fillers of 3 M Filtek Universal and 3 M Filtek Bulk, caused cracks, larger holes from filler removal, and spaces between the filler and resin in resin composites with a regular texture. This resulted in lower wear resistance compared to G-aenial Universal, although the difference was not significant. This can be explained by the fact that these large agglomerates are unable to chemically bond sufficiently with the resin matrix [68] These findings are consistent with those of Checchi V. et al. (2024) [69], who concluded that, following chewing simulation, G-aenial Universal could be an acceptable alternative to paste composites in occlusal regions due to similar wear values. The results also align with Lassila L. et al. (2023) [70], who suggested that G-aenial Universal is a satisfactory alternative for CAD/CAM composite repairs in terms of wear behavior. Additionally, the research backed up the findings of Rajabi H. et al. (2024) [63], who indicated that G-aenial Universal might be appropriate for areas that bear chewing pressure after 200,000 cycles in a wear machine.

In the current study, immersion in acidic solutions, thermal cycling, and wear simulation were done utilizing independent methodologies. So, combining wear modeling, exposure to an acidic solution, and temperature changes offers a better and more effective method for mimicking the real-life conditions that resin composites face in the mouth. In this study, the composites were aged with an acidic solution and thermally cycled for cycles equivalent to one year. However, extending the thermal cycling period beyond one year might provide useful insights into the potential behavior of resin composites under protracted temperature fluctuations; therefore, these in vitro findings require further investigation to assess their intraoral effects.

## Conclusions

Under the conditions of this in vitro study, results suggest that G-aenial Universal Injectable may be the restoration material of choice for neutral oral environments, while 3 M Filtek Z350 XT Universal offers enhanced resistance under acidic conditions; moreover, incremental composites out performed bulkfill ones in surface integrity. Additionally, simulated gastric acid caused more damage than citric acid, which is backed by earlier studies that showed a significant increase in roughness and a decrease in hardness after exposure to gastric acid. These results highlight the importance of selecting restorative materials tailored to patients’ oral chemistry. However, they remain limited to laboratory conditions; clinical validation through long-term in vivo studies is essential before these recommendations can be confidently applied to dental practice.

## Data Availability

The datasets generated and/or analyzed during the current study are not publicly available but are available from the corresponding author upon reasonable request.
